# Changes in Cardiopulmonary Capacity Parameters after Surgery: A Pilot Study Exploring the Link between Heart Function and Knee Surgery

**DOI:** 10.3390/jfmk9030172

**Published:** 2024-09-22

**Authors:** Andrea Segreti, Chiara Fossati, Luigi Maria Monticelli, Daniele Valente, Dajana Polito, Emiliano Guerra, Andrea Zampoli, Giorgio Albimonti, Biagio Zampogna, Sebastiano Vasta, Rocco Papalia, Raffaele Antonelli Incalzi, Fabio Pigozzi, Francesco Grigioni

**Affiliations:** 1 Cardiology Unit, Fondazione Policlinico Universitario Campus Bio-Medico, Via Alvaro del Portillo, 200-00128 Roma, Italy; luigim.8917@gmail.com (L.M.M.); daniele.valente@unicampus.it (D.V.); dajana.polito@unicampus.it (D.P.); f.grigioni@policlinicocampus.it (F.G.); 2 Research Unit of Cardiovascular Science, Department of Medicine and Surgery, Università Campus Bio-Medico di Roma, Via Alvaro del Portillo, 21-00128 Roma, Italy; 3 Department of Movement, Human and Health Sciences, University of Rome “Foro Italico”, Piazza Lauro de Bosis, 15-00135 Roma, Italy; chiara.fossati@uniroma4.it (C.F.); s.vasta@policlinicocampus.it (S.V.); fabio.pigozzi@uniroma4.it (F.P.); 4 Cardiology Division, Department of Biomedical, Metabolic and Neural Sciences, University of Modena and Reggio Emilia, Policlinico di Modena, Via del Pozzo, 71-41124 Modena, Italy; emilianoguerra27@gmail.com; 5 Research Unit of Orthopaedic and Trauma Surgery, Department of Medicine and Surgery, Università Campus Bio-Medico di Roma, Via Alvaro del Portillo, 21-00128 Roma, Italy; andrea.zampoli@unicampus.it (A.Z.); giorgio.albimonti@unicampus.it (G.A.); b.zampogna@policlinicocampus.it (B.Z.); r.papalia@policlinicocampus.it (R.P.); 6 Research Unit of Orthopaedic and Trauma Surgery, Fondazione Policlinico Universitario Campus Bio-Medico, Via Alvaro del Portillo, 200-00128 Roma, Italy; 7 Research Unit of Geriatrics, Department of Medicine and Surgery, Università Campus Bio-Medico di Roma, Via Alvaro del Portillo, 21-00128 Roma, Italy; r.antonelli@policlinicocampus.it; 8 Operative Research Unit of Internal Medicine, Fondazione Policlinico Universitario Campus Bio-Medico, Via Alvaro del Portillo, 200-00128 Roma, Italy

**Keywords:** cardiopulmonary exercise testing, myocardial work, deconditioned amateur athletes, knee injury, surgery

## Abstract

**Background**: A knee injury in an athlete leads to periods of forced exercise interruption. Myocardial work (MW) assessed by echocardiographic and cardiopulmonary exercise testing (CPET) are two essential methods for evaluating athletes during the period following injury. However, compared to pre-surgery evaluations, the variations in cardiovascular parameters and functional capacity assessed by these methods after surgery remain unclear. **Methods**: We evaluated 22 non-professional athletes aged 18–52, involved in prevalently aerobic or alternate aerobic/anaerobic sports activities, who were affected by a knee pathology requiring surgical treatment. The evaluation was performed at rest using transthoracic echocardiography, including MW assessment, and during exercise using CPET. Each athlete underwent the following two evaluations: the first before surgery and the second after surgery (specifically at the end of the deconditioning period). **Results**: Resting heart rate (HR) increased significantly (from 63.3 ± 10.85 to 71.2 ± 12.52 beats per minute, *p* = 0.041), while resting diastolic and systolic blood pressure, forced vital capacity, and forced expiratory volume in the first second did not show significant changes. Regarding the echocardiographic data, global longitudinal strain decreased from −18.9 ± 1.8 to −19.3 ± 1.75; however, this reduction was not statistically significant (*p* = 0.161). However, the global work efficiency (GWE) increased significantly (from 93.0% ± 2.9 to 94.8% ± 2.6, *p* = 0.006) and global wasted work (GWW) reduced significantly (from 141.4 ± 74.07 to 98.0 ± 50.9, *p* = 0.007). Additionally, the patients were able to perform maximal CPET at both pre- and post-surgery evaluations, as demonstrated by the peak respiratory exchange ratio and HR. However, the improved myocardial contractility (increased GWE and decreased GWW) observed at rest did not translate into significant changes in exercise parameters, such as peak oxygen consumption and the mean ventilation/carbon dioxide slope. **Conclusions**: After surgery, the athletes were more deconditioned (as indicated by a higher resting HR) but exhibited better resting myocardial contractility (increased GWE and reduced GWW). Interestingly, no significant changes in exercise capacity parameters, as evaluated by CPET, were found after surgery, suggesting that the improved myocardial contractility was offset by a greater degree of muscular deconditioning.

## 1. Introduction

An athlete is an individual, either young or adult, amateur or professional, engaged in regular exercise training and participating in official sports competitions [1]. The distinction between recreational and competitive athletes is based on the purpose of training: either for pleasure and leisure activities or with a strong emphasis on performance and winning [2]. A proposed classification assesses the minimum volume of exercise in terms of hours per week. Based on this, ‘elite’ professional athletes typically exercise more than 10 h per week, ‘competitive’ amateur athletes more than 6 h per week, and ‘recreational’ amateur athletes more than 4 h per week [3].

Unfortunately, an athlete’s career inevitably involves periods of planned inactivity or forced interruption (e.g., due to illness or injury), which decreases cardiorespiratory and muscular efficiency [4]. These periods of functional rest are characterized by physical deconditioning, i.e., impaired muscle function, cardiovascular efficiency, and global performance [4,5,6]. In the case of a knee injury, athletes frequently require surgical correction to resolve a meniscal or ligament tear [7,8].

In a published paper by our research group, we demonstrated that MW and CPET allow for a comprehensive, non-invasive assessment of injured athletes before knee surgery [9]. We also showed that MW indices, measured at rest, could predict exercise capacity as evaluated by CPET. However, the variations in CPET and MW parameters after knee surgery in athletes have yet to be thoroughly investigated.

## 2. Materials and Methods

The study protocol was approved by the Ethics Committee of Campus Bio-Medico University of Rome (protocol code CPET-INJ, registration number 2022.028). In this non-randomized observational cross-sectional study, we included non-professional athletes aged 18 to 52 with clinical signs or symptoms of knee injury who were candidates for surgical repair of anterior cruciate ligament (ACL) or meniscal tears during a recruitment period lasting approximately two years. The indication for surgery was based on a thorough evaluation of the patient’s medical history, MRI imaging, and physical examination. Competitive non-professional athletes were defined as amateur athletes who trained for more than 6 h per week [3]. Enrolled subjects were required to have a pre-injury exercise time ≥ 6 h per week in one of the following categories of sporting activity envisaged by Dal Monte classification: [10] (a) activities with alternate aerobic/anaerobic exercise; (b) prevalently aerobic activities.

We excluded subjects who were unable to perform CPET due to orthopedic issues (i.e., in the acute phase of the injury), those with concomitant cardiorespiratory comorbidities or acute illnesses, and those with smoking habits (including traditional cigarettes, electronic cigarettes, or heated tobacco products).

Written informed consent was obtained from every patient enrolled in the study [11], and clinical history, physical examination, transthoracic echocardiogram, spirometry, and CPET data were collected.

**Figure 1:**
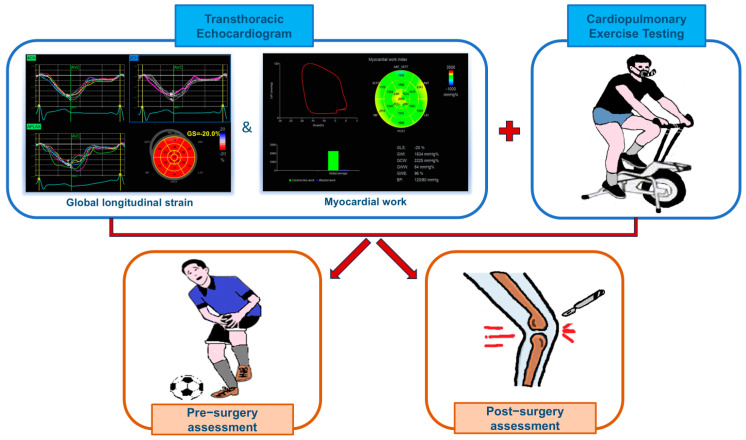
Assessment of the subjects included in the study. The evaluations of the amateur athletes included in the study were performed both pre-surgery and after surgery. The evaluations included advanced echocardiography and cardiopulmonary exercise testing.

All tests were performed in the same time slot (i.e., in the afternoon), and subjects were instructed not to engage in intense training on the same day of the examination and to avoid consuming drinks such as coffee, tea, and chocolate in the 3 h preceding the test. The amateur athletes were assessed both pre-surgery and post-surgery with resting echocardiography, including global longitudinal strain (GLS) and myocardial work (MW), as well as during exercise using cardiopulmonary exercise testing (CPET). Typically, the pre-operative evaluation was conducted 1–2 months after the injury, while the post-operative evaluation occurred 1 to 3 months after surgery, specifically when the athletes were deemed ready to return to exercise using a cycle. Figure 1 illustrates how the athletes were assessed in this study.

### 2.1. Evaluation by Transthoracic Echocardiogram with Myocardial Work Assessment

Each enrolled athlete underwent a transthoracic color-Doppler echocardiogram (GE Vivid T8 and T9, GE Healthcare, General Electric Healthcare, Horten, Norway). Data were obtained from standard two-, three-, and four-chamber apical views at similar HRs, depths, and a frame between 38 and 80 frames/s [12,13]. In addition, a brachial cuff at the time of examination was used to evaluate blood pressure as a non-invasive surrogate of left ventricular (LV) pressure (mmHg).

A post-processing analysis was performed using offline dedicated software (Automated Functional Imaging; EchoPAC^®^, Version 202, General Electrics) to obtain segmental strain (%) and MW parameters [12,13]. A 17-segment model was considered to calculate the segmental MW. Specifically, the LV pressure–strain loop integrated blood pressure (mmHg) with speckle tracking echocardiography (STE) (i.e., strain) throughout the cardiac cycle [14]. The timing of valvular events (i.e., from mitral valve closure to mitral valve opening) was derived using ECG-triggering and Doppler echocardiography [12,13].

Through this technique, it is possible to obtain parameters that describe the relationship between positive and negative myocardial contractions according to their occurrence during the cardiac cycle. Specifically, these parameters include the global work index (GWI), global constructive work (GCW), global wasted work (GWW), and global work efficiency (GWE) [14].

**Figure 2:**
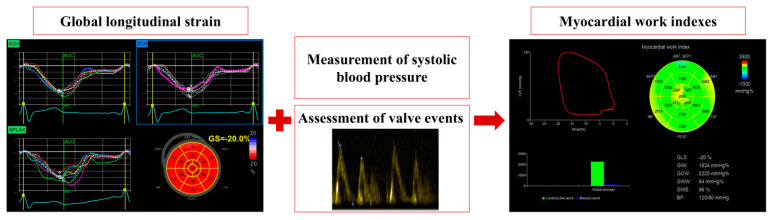
Myocardial work indices. The process to obtain myocardial work indices includes the following: (1) the calculation of the global longitudinal strain from two-, three-, and four-chamber views; (2) measurements of the systolic brachial blood pressure as a non-invasive surrogate of left ventricle systolic pressure; (3) the assessment of valve events. Adapted from Segreti et al. [15].

The GWI (mmHg%) is derived from the average of all segmental MW values and represents the amount of LV work during systole, i.e., from mitral valve closure to mitral valve opening. The GCW (mmHg%) refers to the positive segmental work performed during myocardial systole (shortening) and the negative segmental work during isovolumetric relaxation (lengthening). The GWW (mmHg%) indicates the negative segmental work performed during systole (lengthening) and the positive segmental work performed during isovolumetric relaxation (shortening). Finally, the GWE (%) represents the relationship between constructive work and the sum of constructive and wasted work (i.e., total work). Figure 2 illustrates the process to obtain the MW indices.

### 2.2. Spirometry and Cardiopulmonary Exercise Testing

Every enrolled individual underwent pulmonary function tests (Quark PFT, Cosmed, Albano Laziale, Rome, Italy), including forced vital capacity (FVC) and vital capacity (VC) maneuvers, as outlined by the ATS/ERS Task Force guidelines on the standardization of spirometry [16].

CPET was performed according to the international procedural recommendations of the ARTP guidelines [17]. Each patient pedaled on a cycle ergometer (Quark CPET, Cosmed, Albano Laziale, Rome, Italy), following a custom ramp exercise protocol with a progressive workload lasting approximately 10 min (within a time frame of 8–12 min) to achieve a maximal workload until clinical exhaustion or the onset of factors contraindicating continued exercise [18]. The ramp protocol was calculated based on the Hansen–Wassermann equation, incorporating patient-specific variables such as age, gender, height, and weight to ensure individualized workload progression [19].

Oxygen consumption (VO_2_), carbon dioxide production (VCO_2_), and ventilation (V_E_) were collected breath-per-breath and analyzed using standard techniques [17,18,20]. Other parameters recorded during the test included the heart rate (HR), systolic and diastolic blood pressure (SBP and DBP), work rate (WR), respiratory rate (RR), and peripheral oxygen saturation (SpO_2_).

Peak exercise values were calculated as the average value over a 20-second interval. The anaerobic threshold (AT) was identified through the V-slope analysis of VO_2_ and VCO_2_, and confirmed by the trend analysis of V_E_ vs. VO_2_ (V_E_/VO_2_), V_E_ vs. CO_2_ (V_E_/VCO_2_), end-tidal O_2_ pressure (P_ET_O_2_), and end-tidal CO_2_ pressure (P_ET_CO_2_) [21]. The respiratory compensation point (RCP) was identified by an increase in the V_E_/VCO_2_ ratio and confirmed by a simultaneous decrease in P_ET_CO_2_ [22]. Peak exercise corresponded to the observed VO_2_ max value. The respiratory exchange ratio (RER) was calculated as the ratio between VCO_2_ and VO_2_, and the V_E_/VCO_2_ slope was calculated from the start of exercise up to the RCP.

### 2.3. Statistical Analysis

Unless otherwise stated, all of the values are reported as mean and standard deviation (SD). Statistical analysis was performed using the SPSS 26 software (SPSS Inc, Chicago, IL, USA). We evaluated the changes in echocardiographic and CPET-derived parameters at two evaluation points (i.e., pre-surgery and post-surgery). The Wilcoxon paired test was used to detect differences between pre- and post-surgery values. Results were considered statistically significant for *p*-values < 0.05.

## 3. Results

We evaluated 22 amateur athletes with knee injuries who underwent orthopedic surgery (due to ACL reconstruction or meniscal tears). The demographic characteristics of the enrolled subjects are reported in Table 1

Compared to the pre-surgery evaluation, GLS showed a trend toward reduction, although not statistically significant (from −18.9 ± 1.8 to −19.3 ± 1.75, *p* = 0.161). However, global work efficiency (GWE) increased significantly (from 93.0% ± 2.9 to 94.8% ± 2.6, *p* = 0.006), while global wasted work (GWW) reduced significantly (from 141.4 ± 74.07 to 98.0 ± 50.9, *p* = 0.007) (Table 2).

Compared to the pre-surgery evaluation, the percent-predicted FVC (FVC%) and the percent-predicted FEV_1_ (FEV_1_%) did not significantly change after surgery (Table 3). However, we found that the resting HR increased significantly (from 63.3 ± 10.85 to 71.2 ± 12.52 beats per minute, *p* = 0.041).

The mean ramp protocol used for the pre-surgery CPET was 20.1 ± 4.49, and the same ramp was maintained for the post-surgery CPET. In both pre- and post-surgery evaluation, patients were able to perform a maximal CPET, as demonstrated by the peak RER (1.16 ± 0.15 and 1.16 ± 0.18, respectively) and peak HR (165.09 ± 15.19 and 162.95 ± 16.92, respectively) (Table 3). The mean peak WR, the percent-predicted peak O_2_ consumption (peak VO_2_%), the mean RER, the peak breathing reserve, and the mean ventilation/carbon dioxide (V_E_/VCO_2_) slope did not significantly change (Table 3).

## 4. Discussion

The most common orthopedic sports-related knee injuries involve the meniscus or ligaments, often occurring together [7,23]. These injuries can be treated through repair or reconstruction via arthroscopic or traditional surgery [8]. A knee injury provides an ideal model for evaluating the pulmonary and cardiovascular effects of decreased physical activity in athletes [5]. Following an injury, a comprehensive assessment using advanced echocardiography and CPET offers valuable insight into cardiovascular efficiency, helping to determine the optimal timing for returning to sports and preventing further injuries [9].

CPET is the most comprehensive method for objectively and non-invasively assessing the systems involved in exercise. It identifies the cause of impaired exercise capacity and has applications ranging from advanced heart failure to sports medicine [15,17,24]. It is particularly valuable for functional evaluation in athletes, especially in the various phases following an injury [25,26,27,28].

Transthoracic echocardiography is another pivotal non-invasive tool in sports medicine for evaluating cardiac function [29]. GLS by STE assesses myocardium deformation and is considered a more sensitive indicator of LV systolic function than traditional measures of LVEF [30]. However, the evaluation of 2D-derived GLS in endurance athletes at rest gave contrasting results because of multiple factors, such as heart remodeling, preload, afterload, LV geometry, and sinus bradycardia [31,32,33]. As a result, MW assessment, a new echocardiographic application, has been introduced to quantify global and regional myocardial contractility and to provide insights into cardiac energetics and O_2_ consumption [14,34]. MW is derived non-invasively using LV pressure–strain loop (PSL) analysis, which integrates the non-invasive estimation of LV pressure (using brachial blood pressure by a cuff) and strain analysis by STE [14]. Therefore, MW represents a more reliable measure of myocardial contractility in athletes than traditional echocardiographic measurements, as it overcomes the limitations of other techniques related to heart remodeling [12,13,33,35]. Furthermore, we have previously demonstrated that MW assessment can predict exercise capacity in athletes, as determined by CPET [9,33,36].

Thus, the ability to assess an injured athlete pre-surgery and during various phases post-surgery using non-invasive, rapid, and comprehensive techniques like MW and CPET can provide crucial insights for developing a structured program to identify the optimal time for returning to sport, improve overall performance, and reduce the risk of future injuries [9].

In the present study, we observed a significant increase in resting HR after surgery, suggesting that athletes were more deconditioned at this stage. Indeed, highly trained athletes typically exhibit increased parasympathetic tone, which leads to a lower resting HR [15].

At the same time, we observed significant improvements in resting MW parameters post-surgery compared to pre-surgery. Specifically, there was a statistically significant decrease in the GWW, which represents negative myocardial work, i.e., the negative segmental work performed during systole (lengthening) and positive segmental work performed during myocardial isovolumic relaxation (shortening). Additionally, we found a significant increase in the GWE, which reflects the relationship between constructive work and the sum of constructive and wasted work (i.e., total work).

In this study, we also evaluated differences in exercise capacity. In a previous study conducted by our research group, we demonstrated that athletes requiring surgical treatment for ACL or meniscal tears could successfully perform a maximal CPET on a cycle ergometer [9]. In that study, we evaluated 28 non-professional athletes aged 18–52, involved in prevalently aerobic or alternate aerobic/anaerobic sports activities, who had knee pathologies necessitating surgical treatment. Evaluations were performed at rest using transthoracic echocardiography, which included the assessment of GLS and MW, and during exercise using CPET. Despite the pain associated with knee injuries, patients were able to complete maximal effort stress testing, as CPET via cycle ergometer has been shown to be well-tolerated by this patient group.

In the present study, we found that the percent-predicted peak VO_2_ was 82.8 ± 13.7%, the mean RER was 1.16 ± 0.08, and the mean ventilation/carbon dioxide (VE/VCO_2_) slope was 24.23 ± 3.36. Additionally, peak VO_2_ was negatively correlated with GLS (r = −0.518, *p* = 0.003) and global wasted work (GWW) (r = −0.441, *p* = 0.015) and positively correlated with global work efficiency (GWE) (r = 0.455, *p* = 0.012). Finally, we found that the VE/VCO_2_ slope during exercise was negatively correlated with GWE (r = −0.585, *p* = 0.001) and positively correlated with GWW (r = 0.499, *p* = 0.005) [9].

Another important aspect is that CPET provides essential details on pre-operative risk, allowing for safer surgery and the optimization of surgical procedures [37,38]. This is particularly relevant for master athletes, who often present with cardiac and respiratory comorbidities. Compared to a treadmill, the cycle ergometer offers greater stability for the affected knee, making it easier for patients with knee injuries to exercise. Furthermore, obtaining a baseline assessment is crucial for tracking changes in cardiorespiratory function and performance over time, particularly after surgical treatment. In the present study, all of the athletes were able to perform maximal effort testing on the cycle ergometer, as evidenced by the peak RER values ≥ 1.10 and the maximal predicted HR at a peak exercise of ≥85%.

Explaining a possible link between the knee and the heart is complex. It has been demonstrated that, in the presence of a damaged knee joint, acute local inflammation occurs, possibly followed by synovitis [39]. It has also been shown that local inflammation can lead to systemic inflammation [40]. Mechanical stress applied to a joint can be converted into intracellular signals activated by mechanoreceptors on the surface of joint cells (ion channels and integrins). When a certain threshold level is reached, these signals lead to the overexpression of soluble inflammatory mediators, such as prostaglandins, chemokines, and cytokines. For example, the serum levels of inflammatory cytokines, including IL-6, IL-13, and TNF-α, were significantly elevated in patients with osteoarthritis secondary to meniscus injury [40].

Extensive literature suggests that, regardless of the underlying etiology, inflammatory cytokines are also widely involved in the pathogenesis of heart failure. Inflammatory cytokines modulate the phenotype and function of myocardial cells, suppressing the contractile function in cardiomyocytes, inducing macrophage activation, stimulating inflammation and microvascular dysfunction, and promoting the fibrogenic action of fibroblasts [41]. Furthermore, it has been demonstrated that elevated systemic inflammatory markers are associated with subclinical myocardial dysfunction and abnormal MW indices [42,43].

Therefore, we could speculate that, in the case of a knee injury, inflammatory factors spread beyond the knee compartment, leading to systemic inflammation and compromised myocardial activity. Since MW indices are the most accurate method to capture the consequences of heart damage, even in the subclinical phase, the reversal of inflammation by repairing or reconstructing the knee lesion would explain the improved MW indices found after surgery. The results of the present study indicate that, after surgery, amateur athletes were more deconditioned (higher resting HR values) but had a concomitant increase in the GWE and a reduction in the GWW, suggesting better resting myocardial contractility.

Generally, athletes can achieve a much higher peak VO_2_ value than sedentary individuals due to a higher Qc and greater capacity to transport O_2_ to the muscles [15,27,44]. However, the mean peak VO_2_ value we found in pre- and post-surgery evaluations is undoubtedly lower than those generally found in well-trained individuals because we evaluated a different population (i.e., deconditioned amateur athletes following an injury). Therefore, even though all of the subjects maintained normal involvement in daily activities, particularly before surgery, this reduction in VO_2_ was expected. Knee injury, hospitalization, surgery, and the subsequent resting period undoubtedly led to cardiac deconditioning, characterized by an increased resting HR, reduced LV end-diastolic dimension, decreased CO, and reduced capacity to transport and use O_2_ [45,46,47]. All of these effects translate into a decline in the peak VO_2_.

However, the present study did not find significant differences in post-surgery CPET parameters compared to pre-surgery evaluations. For example, we found no substantial variation in the peak VO_2_, the primary determinant of exercise performance. Although athletes were deconditioned in both evaluations, a further reduction in exercise capacity, specifically in peak VO_2_ after surgery, would be expected compared to the pre-surgery assessment. According to Fick’s equation, we must assume that VO_2_ equals cardiac output (Qc) times arteriovenous oxygen difference, C(a−v)O_2_. Therefore, VO_2_ determinants are cardiac, pulmonary, and peripheral (muscular) factors [15]. All of the included patients had normal respiratory function values in both evaluations, as determined by spirometry. We also did not find significant differences in other respiratory and pulmonary vascular parameters, such as the breathing reserve and VE/VCO_2_ slope. Therefore, it is possible that the VO_2_ did not change because, after surgery, cardiac function increased, at least in resting conditions, while the patients remained more deconditioned. This suggests that the improvement in central determinants (increased GWE and reduced GWW) was counterbalanced by a worsening of the peripheral components induced by deconditioning. This balance between these determinants explains why peak VO_2_ remained almost unchanged.

### Limitations

Each participant was deconditioned, as reported by the patients and demonstrated by the CPET parameters. However, the time elapsed from injury to the pre-surgery evaluation, and, consequently, the degree of deconditioning, differed among the enrolled subjects. To minimize this variation, we evaluated the patients as soon as possible after the first orthopedic evaluation (before surgery), avoiding the enrollment of patients in the acute phase of injury (at least 15 days post-injury). Additionally, we enrolled patients with similar post-operative restrictions and rehabilitation protocols.

Some patients may have reduced their exercise capacity to avoid knee pain during CPET. However, the cycle ergometer is one of the most tolerated exercise modalities by patients with a knee injury. All of the patients enrolled in the present study reached a peak RER ≥ 1.10, indicating maximal effort in both evaluations.

We assumed that the peak VO_2_ did not significantly change after exercise due to a balance between improved myocardial contractility and reduced physical conditioning. However, a more complex CPET would be recommended to test this hypothesis effectively, evaluating several determinants of peak VO_2_, such as the non-invasive assessment of cardiac output and peripheral muscular O_2_ extraction.

Finally, considering the nature of this study as a pilot, the population was not stratified according to age and gender, which may have an impact on the MW indices [48].

## 5. Conclusions

The results of the present study have practical implications for the post-surgery rehabilitation of athletes. We found that resting myocardial contractility indices improved after the surgical repair of the knee lesion. However, the athletes were more deconditioned, suggesting a possible link between the reversal of knee inflammation and improved myocardial contraction. In this context, MW proved capable of detecting even subclinical myocardial dysfunction, which seemed to regress after surgery. We also postulated that systemic inflammatory factors could be responsible for subclinical myocardial damage. However, the results of the present pilot study should be confirmed by determining local and systemic inflammatory markers.

## Figures and Tables

**Table 1:** Demographic characteristics and resting vital signs of the enrolled subjects.

Characteristic	Total, *n* = 22
Age, years, mean ± SD	37.9 ± 12.9
Male sex, *n* (%)	19 (86.4)
Height, mean ± SD	173.3 ± 7.0
Weight, mean ± SD	73.6 ± 12.2
Body Mass Index, mean ± SD	24.4 ± 3.4

All of the values are reported as mean ± SD.

**Table 2:** Echocardiographic parameters obtained before and after knee surgery.

Echo Parameters	Pre-Surgery *n* = 22	Post-Surgery *n* = 22	*p*-Value
**GLS (%)**	18.9 ± 1.8	19.3 ± 1.7	0.161
**GWI (mmHg%)**	1685.9 ± 153.8	1702.1 ± 163.1	0.698
**GWE (%)**	92.4 ± 3.1	95.1 ± 2.1	0.006
**GCW (mmHg%)**	2127.7 ± 194.7	2069.5 ± 209.9	1.000
**GWW (mmHg%)**	141.4 ± 74.1	98.0 ± 50.9	0.007

All values are reported as mean ± SD. GLS = global longitudinal strain; GWI = global work index; GWW = global wasted work; GCW = global constructive work; GWE = global work efficiency.

**Table 3:** Cardiopulmonary capacity parameters obtained before and after knee surgery.

CPET Parameters	Pre-Surgery *n* = 22	Post-Surgery *n* = 22	*p*-Value
**HR rest (bpm)**	63.3 ± 10.8	71.2 ± 12.5	0.041
**SAP rest (mmHg)**	118.2 ± 9.8	118.6 ± 9.8	0.848
**DAP rest (mmHg)**	75.9 ± 6.7	76.9 ± 8.7	0.591
**SpO_2_ rest (%)**	98.1 ± 0.8	97.9 ± 0.9	0.361
**FVC% ° (%)**	105.1 ± 11.1	104.7 ± 13.5	0.408
**FEV_1_% ^§^ (%)**	106.2 ± 10.7	105.7 ± 11.8	0.277
**MVV (L/min)**	169.9 ± 33.1	169.0 ± 34.3	0.618
**HR max (bpm)**	165.1 ± 15.2	163.0 ± 16.9	0.323
**SAP max (mmHg)**	157.5 ± 20.5	166.3 ± 22.5	0.771
**DAP max (bpm)**	88.6 ± 8.9	84.7 ± 10.3	0.186
**Power (Watt)**	170.1 ± 38.6	159.0 ± 41.5	0.154
**Peak RER**	1.16 ± 0.07	1.16 ± 0.08	0.980
**Peak VO_2_% ^ç^ (%)**	84.14 ± 14.7	84.9 ± 15.6	0.739
**V_E_/VCO_2_ slope**	24.5 ± 3.0	25.4 ± 4.1	0.185
**BR (%)**	51.4 ± 11.2	51.3 ± 9.9	0.973
Peak O_2_ pulse * (mL/beat)	13.5 ± 2.7	13.9 ± 2.6	0.748
**VO_2_/WR slope (mL/min/Watt)**	10.6 ± 1.0	10.6 ± 1.3	0.908

All values are reported as mean ± SD. BR = breathing reserve; CPET = cardiopulmonary exercise testing; DAP = diastolic arterial pressure; HR = heart rate (beats per minute); O_2_ = oxygen; FEV_1_ = forced expiratory volume in the first second; FVC = forced vital capacity; MVV = maximal voluntary ventilation; RER = respiratory exchange ratio; SAP = systolic arterial pressure, SpO_2_ = oxygen saturation; V_E_/VCO_2_ = ventilatory equivalent for carbon dioxide; VO_2_ = oxygen uptake. ^§^ FEV_1_% = percent-predicted forced expiratory volume in the first second; ° FVC% = percent-predicted forced vital capacity; ^ç^ Peak VO_2_% = percent-predicted peak oxygen consumption; * O_2_ pulse = VO_2_/HR.

## Data Availability

The data presented in this study are available upon request.
